# Embryo thermal manipulation modifies development and hepatic lipid metabolism in post-hatch layer-type chicks

**DOI:** 10.1093/jas/skae242

**Published:** 2024-08-21

**Authors:** Sheng Li, Yuyan Wang, Siyu Guo, Xiaoqing Li, Guofeng Han, Zilin Zhou, Chunmei Li

**Affiliations:** Research Centre for Livestock Environmental Control and Smart Production, College of Animal Science and Technology, Nanjing Agricultural University, Nanjing 210095, China; Research Centre for Livestock Environmental Control and Smart Production, College of Animal Science and Technology, Nanjing Agricultural University, Nanjing 210095, China; Research Centre for Livestock Environmental Control and Smart Production, College of Animal Science and Technology, Nanjing Agricultural University, Nanjing 210095, China; Research Centre for Livestock Environmental Control and Smart Production, College of Animal Science and Technology, Nanjing Agricultural University, Nanjing 210095, China; Research Centre for Livestock Environmental Control and Smart Production, College of Animal Science and Technology, Nanjing Agricultural University, Nanjing 210095, China; Research Centre for Livestock Environmental Control and Smart Production, College of Animal Science and Technology, Nanjing Agricultural University, Nanjing 210095, China; Research Centre for Livestock Environmental Control and Smart Production, College of Animal Science and Technology, Nanjing Agricultural University, Nanjing 210095, China

**Keywords:** thermal manipulation, lipid metabolism, layer-type chicken, liver, hatching

## Abstract

Incubation temperature is a crucial environmental factor affecting embryonic development and chick quality. Metabolism during the embryonic stage, particularly liver lipid metabolism, is essential for the growth and development of poultry. This study aimed to investigate the effects of embryo thermal manipulation with high (**TMH**, 39.5 °C, 65% RH, 8 h/d) and low (**TML**, 20 °C, 65% RH, 1 h/d) temperatures during 8th to 15th embryonic age on hatching performance and liver lipid metabolism in layer chicks. Additionally, the duration of TM effects was evaluated through a short-term feeding trial. The results indicated that TMH accelerated the hatching process without significantly affecting hatchability and growth performance. In contrast, TML delayed hatching time and significantly reduced hatchability and chick quality. After hatching, TML also increased residual yolk weight and reduced the relative liver weight in relation to body weight and yolk-free body mass. Moreover, lipid droplets in the liver were stained with Oil Red O, and the lipid content in the liver and serum was further detected. TMH had no significant impact on triglyceride (TG) and total-cholesterol (TCHO) content in the liver and serum but upregulated the expression of lipogenesis-related genes *ACC*, *Fas*, and *Fatp1* compared to the TML group. Conversely, TML significantly reduced liver TG content, enhanced lipoprotein lipase (LPL) activity, and promoted the expression of lipid oxidation-related genes *CPT-1*, *PGC-1α*, and *PPARα*. At 7 d of age, liver LPL activity was significantly increased in the TMH group. However, there were no significant changes in the content of TG and TCHO in the liver and the expression of lipid metabolism-related genes in the TML group. Overall, these results indicate that embryonic TM alters hatching performance and liver lipid metabolism in layer chicks. TML reduces TG content by increasing liver lipid oxidation capacity. However, this effect is not long-lasting, as the influence of TM diminishes as the chicks develop.

## Teaser

Embryo thermal manipulation with low temperature decreases hepatic triglyceride content by enhancing lipolysis and lipid oxidation in post-hatching layer chicks.

The effects of embryo thermal manipulation on performance and hepatic lipid metabolism diminish as the chicks develop.

## Introduction

Incubation temperature is a critical factor influencing the hatching quality and physiological status of chickens. Changes in incubation temperature during the embryonic stage, also known as thermal manipulation (**TM**), have been reported to alter post-hatch development and environmental adaptation of chicks, with long-term effects (Al-Zghoul and El-Bahr, 2019; Goel et al., 2023; Xu et al., 2023). Although the impact of TM on the development of chicken body remains a subject of debate, it largely hinges on whether the incubation temperature is higher or lower than the standard. Generally, an incubation temperature higher than the standard (37.5 °C) is beneficial for shortening the incubation period and increasing hatching weight (Al-Zghoul and El-Bahr, 2019; Amaz et al., 2024). However, excessively high temperatures (39.6 °C, 60% RH) can negatively affect the quality and performance of chicks after hatching (Narinç et al., 2016). Furthermore, short-term cold exposure (15 °C lasting 30 or 60 min) is thought to improve post-hatch broiler thermoregulation and cardiovascular system adaptation to cold conditions (Shinder et al., 2011), although few studies have reported positive effects of this strategy on development (Nyuiadzi et al., 2017, 2020). For instance, chicks hatched at a lower temperature (35.5 °C) exhibited the longest development time and remained smaller at hatch and post-hatch compared to those hatched under normal conditions (Nord and Nilsson, 2021). It is speculated that this growth regulatory effect is more attributable to nutritional and metabolic status during the embryonic period or after hatching (Goel et al., 2023).

Most nutrients during embryonic development are supplied by the yolk, with lipid utilization being crucial for the growth and development of chicken embryos. During incubation, approximately 50% of the total yolk fatty acids are incorporated into embryonic tissue (Speake et al., 1998). The remaining fatty acids undergo beta-oxidation to produce saturated fatty acids to provide energy for development (van der Wagt et al., 2020). The liver is the primary tissue of lipid metabolism in poultry, converting yolk fatty acids into a form that the embryo can utilize (Noble and Cocchi, 1990). After hatching, a de novo fat synthesis pathway in the liver uses non-lipid substances as substrates to synthesize triglycerides, which are then transported to adipose tissue for storage via the bloodstream (Nematbakhsh et al., 2021). Additionally, the liver is the main organ for lipid oxidation and utilization. Triglycerides are hydrolyzed by lipolytic enzymes to produce fatty acids, which are taken up into mitochondria through carnitine palmitoyltransferase-1 (CPT-1) for oxidative phosphorylation to provide energy (Schlaepfer and Joshi, 2020). TM has limited effects on lipid metabolism in embryonic ducks but promotes fat deposition during the post-hatch growth stage (Wang et al., 2014). However, some studies indicate that high incubation temperatures reduce lipid and carbohydrate metabolism, resulting in impaired egg development and smaller chicks (Willemsen et al., 2011). Although current evidence suggests that the TM process involves the regulation of lipid metabolism, it remains unclear whether ambient temperature, particularly below standard incubation temperatures, affects lipid metabolism homeostasis in the liver.

Laying hens exhibit high lipid metabolism activity, making lipid metabolism disorders and subsequent conditions like fatty liver significant challenges in performance. However, the potential benefits of TM treatment in layer chicks have received less attention. The objective of this study was to investigate the effects of periodic heat or cold exposure during the embryonic period on chick quality and liver lipid metabolism in commercial layer-type strains. Additionally, the duration of the regulatory effects of TM on early liver lipid metabolism was evaluated through a short-term feeding experiment.

## Materials and Methods

All research procedures were approved by the Nanjing Agricultural University Animal Care and Use Committee (Permit Number SYXK (Su) 2017-0007) and complied with the Regulations on the Administration of Laboratory Animals promulgated by the National Science and Technology Commission of the People’s Republic of China (Beijing).

### Animals and treatment

The fertile eggs (Hy-Line Variety Brown) were purchased from a hatchery in Jiangsu, China, and incubated at standard temperature and humidity (37.5 °C, 65% RH) until the 7th embryonic age. Eggs that did not exhibit normal development were identified and discarded using the candling method. At the 8th embryonic age, 306 eggs were weighed and randomly divided into 3 groups with 6 replicates per group and 17 eggs per replicate: control (Con, 60.18 ± 0.62 g) group, TM with high temperature (**TMH**, 60.31 ± 0.65 g) group, and TM with low-temperature (**TML**, 60.22 ± 0.60 g) group. During embryonic days (**EDs**) 8 to 15, the hatching temperature of eggs in TMH group was increased from the standard state of incubation (37.5 °C, 65% RH) to the high-temperature state of incubation (39.5 °C, 65% RH) for 8 h every day. The hatching temperature of eggs in TML group was decreased from the standard state of incubation (37.5 °C, 65% RH) to the low-temperature state of incubation (20 °C, 65% RH) for 1 h every day. During the remainder of the incubation period, the temperature and relative humidity for the TMH and TML groups remained consistent with the Con group, which continued to be incubated under standard conditions (37.5 °C, 65% RH) throughout.

After hatching, 54 healthy chicks with similar body weights (**BWs**, approximately 35 ± 1 g) from 3 groups were transferred and housed in environmentally controlled rooms, with 3 replicates per group and 6 birds per replicate. These rooms are equipped with a temperature and humidity sensor, the Zl-th10TP (CIMC Technology Co., Ltd., Beijing, China), to monitor indoor conditions. An intelligent control system (iRVC-045, Kooland, Beijing, China) integrates these temperature and humidity parameters and adjusts the indoor heating and cooling air conditioners, as well as humidifiers, in real-time to maintain conditions within the specified range. The system features a temperature control accuracy of 1 °C and a humidity control accuracy of 7%. During the brooding period from days 1 to 7, the environmental temperature was consistently maintained at 32 ± 1 °C, and the RH was controlled between 50% and 60%. Chicks were fed commercial starter diet (16.5% crude protein and 2,650 kcal/kg of metabolizable energy) until the end of the experiment. Feed was offered ad libitum in mash form, and water was available at all times. The composition of the diet is detailed in Table 1.

**Table 1.** The composition and nutrient levels of the experimental diets (1 to 7 d)

Ingredients, %	1 to 7 d
Corn, 8.5%	59.65
Soybean meal, 46%	22.91
Soybean oil	1.14
Limestone powder	8.93
Wheat bran	5.00
Calcium hydrophosphate	1.53
Salt	0.35
Lysine, 99%	0.04
Methionine, 98%	0.10
Choline chloride, 50%	0.10
Vitamin premix feed^1^	0.05
Trace element feed^2^	0.20
Calculated nutrient composition	
Metabolic energy, kcal/kg	2650
Crude protein, %	16.5
Crude fiber, %	2.6
Calcium, %	3.54
Available phosphorus, %	0.40

^1^Vitamin premix provides the following per kg of diet: VA, 8,000 IU; VD3, 3,000 IU; VE, 20 IU; VK, 2 mg; VB1, 4 mg; riboflavin, 8 mg; D-pantothenic acid, 11 mg; VB5, 40 mg; VB6, 4 mg; VB12, 0.02 mg; biotin, 0.15 mg; folic acid, 1.0 mg; choline, 700 mg.

^2^Mineral premix provides the following per kg of diet: Fe (as ferrous sulfate), 80 mg; Zn (as zinc sulfate), 75 mg; Mn (as manganese sulfate), 80 mg; Cu (as copper sulfate) 10 mg, I (as potassium iodide), 0.40 mg; and Se (as sodium selenite), 0.30 mg.

### Sample collection

At 1 d of age, 12 chicks (both males and females) in each treatment group were randomly selected and euthanized. Euthanasia was conducted through CO_2_ asphyxiation, followed by exsanguination. Then the BW and yolk weight (YW) were measured by an electric balance. The liver, breast muscle, thigh muscle, and heart were harvested and weighed. The liver sample was obtained and immediately frozen in liquid nitrogen for subsequent analysis. Portions (around 200 mg) of the liver was excised and fixed in 4% paraformaldehyde solution for histomorphological observation. The yolk-free body mass (YFBM) was calculated as the difference between BW and YW. The relative weight (%) of the heart, breast muscle, thigh muscle, and liver were calculated as the ratio between the tissue weight and the BW or YFBM.

At 7 d of age, feed intake and BW gain were recorded per cage to calculate feed conversion ratio (**FCR**, ADFI/ADG). After a 12-h overnight fast, 12 chicks (both males and females) in each treatment group were randomly selected and euthanized. The liver, breast muscle, thigh muscle, abdominal fat, and heart were harvested and weighed. The collection of the liver sample is the same as above.

### Hatchability evaluation

At the 18th embryonic age (432 h of incubation), all eggs were transferred to the incubation tray. A video system (HIKVISION, Hangzhou, China) was used to record the incubation process of the embryo eggs until the end of incubation at 504 h. The number of hatched chicks in each group was recorded every 2 h to generate the incubation curve.

### Chick quality evaluation

After hatching, chick quality evaluation was conducted following the methodology of a previous study (Tona et al., 2003). Briefly, the parameters evaluated included activity, down and appearance, retracted yolk, eyes, legs, navel area, remaining membrane, and remaining yolk. Each parameter was assessed and scored based on its importance, contributing to a total score of 100.

### Body temperature measurement

The rectal temperature was assessed using a Thermalert monitoring thermometer (TH-5, Physitemp, Clifton, NJ). The thermometer probe was inserted into the rectum to a depth of 2 to 3 cm, and data from the thermometer were recorded 2 s later. The overall accuracy of the measuring system was ± 0.1 °C.

### Triglycerides and total-cholesterol content

The contents of triglycerides (**TG**) and total-cholesterol (**TCHO**) were determined with commercial kits (Nanjing Jiancheng Bioengineering Institute, Nanjing, China). The content of TG and TCHO in the liver was expressed as a ratio with protein concentration. Total protein concentration of the homogenate was measured by BCA Protein Assay (Beyotime, Shanghai, China) using bovine serum albumin as the standard.

### Liver Oil Red O staining

The measurement of liver lipid droplets was based on that described by the previous research (Zhang et al., 2019). Briefly, tissue sections of the liver were stained with Oil Red O (Solarbio, Beijing, China), and examined under a microscope (BX51, Olympus, Tokyo, Japan) to evaluate the content of lipid droplets.

### Lipase activity and fatty acid synthase content

According to the manufacturer’s instructions, the activities of lipoprotein lipase (**LPL**) and hepatic lipase (**HL**) were measured with commercial diagnostic kits (Nanjing Jiancheng Bioengineering Institute). The content of fatty acid synthase (**Fas**) was determined using commercial ELISA kits. The enzyme activities and Fas content in the liver were expressed as a ratio with protein concentration.

### RT-qRCR

Total RNA was extracted from the liver using TRIzol reagent (Invitrogen, Carlsbad, CA), followed by quantification of RNA concentration via spectrophotometry (Thermo Fisher Scientific, Waltham, MA). Subsequently, reverse transcription was conducted for first-strand cDNA synthesis using the Transcriptor First-Strand cDNA Synthesis Kit (ABclonal, Wuhan, China). The synthesized cDNA was then subjected to amplification in a 20 μL PCR system containing 1 μg of total RNA, 0.2 μmol/L of each specific primer (Sangon, Shanghai, China) and SYBR Green master mix (ABclonal) according to manufacturer’s protocol. Real-time PCR was carried out using an ABI QuantStudio 7 PCR machine (Applied Biosystems; Thermo Fisher Scientific), with the primer sequences outlined in Table 2. The PCR products were verified by electrophoresis and DNA sequencing. The mRNA levels of the target genes were normalized to β-actin (ΔCT).

**Table 2.** PCR primer sequences

Gene	Gene Bank Number *Gallus gallus*	Primer sequences (5ʹ→3ʹ)	Product size, bp
*ACC*	XM_015295697.2	Forward: AATGGCAGCTTTGGAGGTGT	136
		Reverse: TCTGTTTGGGTGGGAGGTG	
*ADP*	XM_015276846.2	Forward: ACCCAGACACAGATGACCGTT	239
		Reverse: GAGCAAGAGCAGAGGTAGGAGT	
*ATGL*	NM_001113291.1	Forward: AAGTCCTGCTGGTCCTCTCCTTG	94
		Reverse: AGTGTTGTCCTCCATCTGGTCCTC	
*AMPKα*	NM_001039605.1	Forward: GGGACCTGAAACCAGAGAACG	215
		Reverse: ACAGAGGAGGGCATAGAGGATG	
*CPT-1*	XM_015286798.2	Forward: GGAGAACCCAAGTGAAAGTAATGAA	135
		Reverse: GAAACGACATAAAGGCAGAACAGA	
*Fas*	NM_205155.3	Forward: CTATCGACACAGCCTGCTCCT	107
		Reverse: CAGAATGTTGACCCCTCCTACC	
*Fatp1*	NM_001039602.2	Forward: TCAGGAGATGTGTTGGTGATGGAT	138
		Reverse: CGTCTGGTTGAGGATGTGACTC	
*FXR*	NM_204113.2	Forward: AGTAGAAGCCATGTTCCTCCGTT	182
		Reverse: GCAGTGCATATTCCTCCTGTGTC	
*LPL*	XM_015280414.2	Forward: CAGTGCAACTTCAACCATACCA	150
		Reverse: AACCAGCCAGTCCACAACAA	
*ME*	NM_204303.1	Forward: TGCCAGCATTACGGTTTAGC	175
		Reverse: CCATTCCATAACAGCCAAGGTC	
*PGC-1α*	XM_046916275.1	Forward: GACTCAGGTGTCAATGGAAGTG	272
		Reverse: ATCAGAACAAGCCCTGTGGT	
*PPARα*	XM_046906400.1	Forward: AGACACCCTTTCACCAGCATCC	167
		Reverse: AACCCTTACAACCTTCACAAGCA	
*PPARγ*	XM_015292933.2	Forward: CCAGCGACATCGACCAGTT	145
		Reverse: GGTGATTTGTCTGTCGTCTTTCC	
*Srebp-1c*	NM_204126.2	Forward: GCCCTCTGTGCCTTTGTCTTC	130
		Reverse: ACTCAGCCATGATGCTTCTTCC	
*β-Actin*	NM_205518.2	Forward: CTGGCACCTAGCACAATGAA	123
		Reverse: CTGCTTGCTGATCCACATCT	

### Statistical analysis

In this study, chicks were sexed at 1 and 7 d of age. Data from male and female chicks were initially analyzed separately to account for any sex-related differences in response to TM. Preliminary statistical analysis indicated that the effects of TM on performance and liver lipid metabolism were consistent across both sexes. Consequently, the primary analysis in this study focused on the overall effects of TM on the chicks, encompassing both males and females, to ensure that the results accurately reflected the treatment effects without the confounding influence of sex differences.

Statistical analysis was conducted using one-way ANOVA with Statistical Analysis System software (version 8e; SAS Institute, Cary, NC), and data were represented as the mean ± SEM. Mean separation was carried out using Tukey’s multiple comparisons, and treatment effects were deemed statistically significant at a probability level of *P* < 0.05.

## Results

### Effect of TM on hatchability and quality of chicks

**Figure 1.**
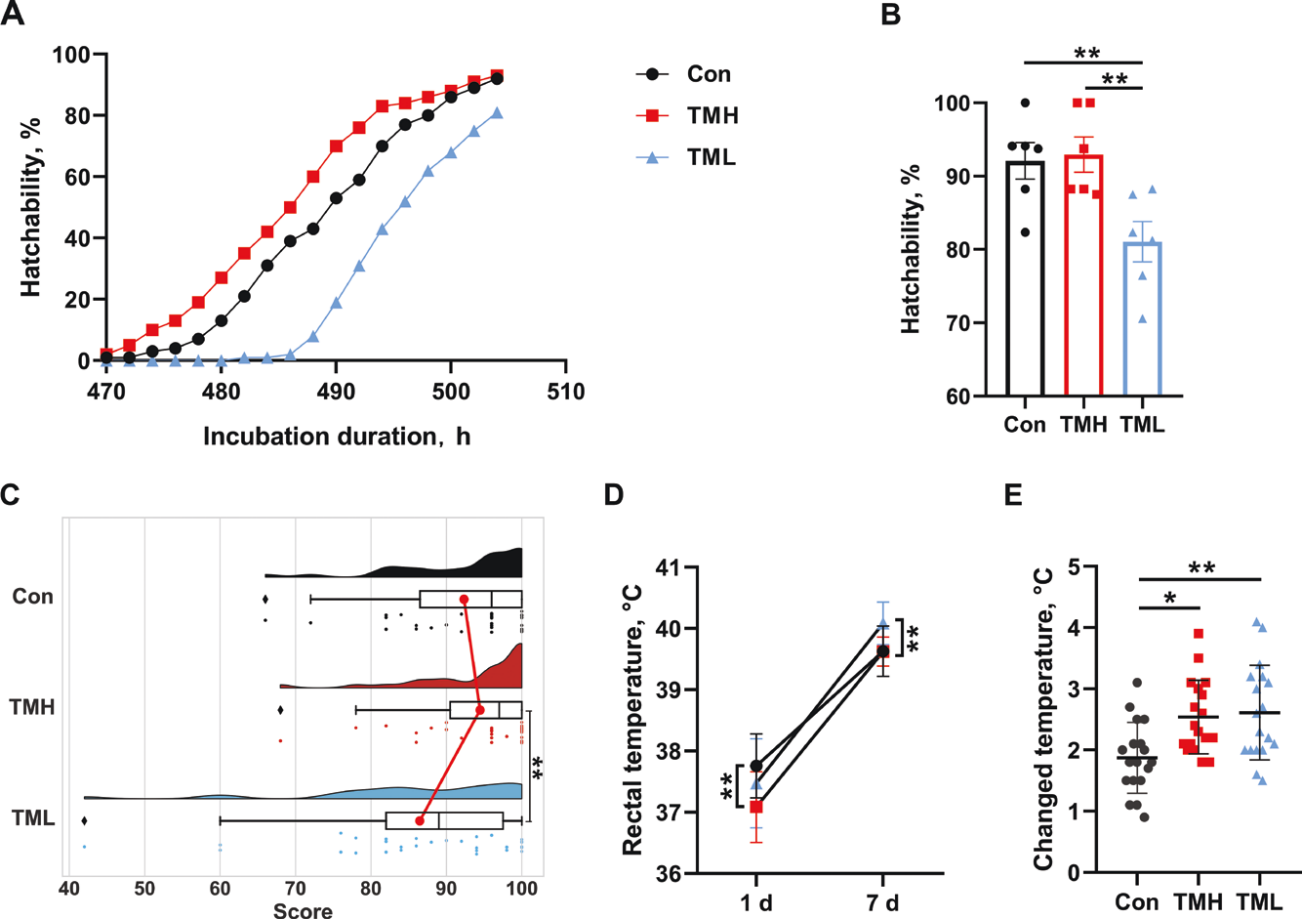
Effect of TM with different temperatures on hatch performance of chicks. (A) Hatching duration; (B) hatchability (*n* = 6); (C) score of chick quality (*n* = 30); (D) rectal temperature of chicks at 1 and 7 d (*n* = 18); (E) change in rectal temperature from 1 to 7 d (*n* = 18). Data are expressed as the mean ± SEM. *P < 0.05, **P < 0.01, ***P < 0.001.

As shown in Figure 1, the incubation duration and hatchability were affected by TM with different temperatures. TMH promoted the hatching process compared to the control group. However, TML delayed egg hatching, and the hatchability was significantly reduced compared to the control and TMH groups (*P* < 0.05). “Tona evaluation” was performed to assess the quality of the chicks. As shown in Figure 1C, there was no significant difference in score of post-hatching chicks between the control and TMH groups (*P* > 0.05). However, chicks from the TML group scored lower than those from the TMH group (*P* < 0.05). The rectal temperature of the TMH-treated group was significantly lower than that of the control group at 1 d of age (*P* < 0.05), while there was no difference in the TML group (*P* > 0.05). However, chicks in the TML group had the highest rectal temperature compared to the other groups at 7 d of age (*P* < 0.05). Additionally, the change in rectal temperature from 1 to 7 d of age in the TMH and TML groups was significantly higher than that in the control group (*P* < 0.05).

### Effect of TM on performance of post-hatch chicks

Compared to the control group, TM had no effect on the BW and body length (**BL**) of post-hatching chicks (Table 3). However, TML-treated chicks had significantly higher residual YW and YW percentage of BW (*P* < 0.05), but a similar YFBM. Moreover, the liver percentage of BW and YFBM in the TML group decreased compared to the other groups (*P* < 0.05), while the thigh muscle percentage of YFBM significantly increased (*P* < 0.05). No notable changes were observed in the relative weight of the heart and breast muscle in either the TMH or TML groups (*P* > 0.05).

**Table 3.** Effect of TM with different temperatures on performance of chicks at 1 d of age

Items	Con	TMH	TML	SEM	*P* value
BW (g)	34.11	35.53	36.50	0.510	0.1568
BL (cm)	14.84	14.93	14.71	0.074	0.4959
YW (g)	3.10^b^	3.13^b^	4.08^a^	0.148	0.0059
YFBM (g)	31.01	32.40	32.43	0.415	0.2879
YW/BW (%)	9.01^b^	8.78^b^	11.07^a^	0.327	0.0041
Liver/BW (%)	2.44^a^	2.36^a^	2.16^b^	0.039	0.0054
Liver/YFBM (%)	2.68^a^	2.58^ab^	2.42^b^	0.039	0.0164
Heart/BW (%)	0.81	0.78	0.84	0.021	0.5294
Heart/YFBM (%)	0.89	0.85	0.94	0.023	0.3118
Breast muscle/BW (%)	1.74	1.93	1.67	0.054	0.1416
Breast muscle/YFBM (%)	1.91	2.11	1.88	0.059	0.2342
Thigh muscle/BW (%)	8.02	8.34	8.80	0.141	0.0754
Thigh muscle/YFBM (%)	8.82^b^	9.16^ab^	9.90^a^	0.169	0.0233

^a,b^Values with different superscripts in the same row differ significantly (*P* < 0.05).

Con, control treatment (37.5 °C) during incubation; TMH, thermal manipulation with higher incubation temperature (39.5 °C and 65% RH for 8 h/d during 8th to 15th embryonic age) during incubation; TML, thermal manipulation with lower incubation temperature (20 °C and 65% RH for 1 h/d during 8th to 15th embryonic age) during incubation; BW, body weight; BL, body length; YW, yolk weight; YFBM, yolk-free body mass.

### Effects of TM on hepatic lipid metabolism of post-hatch chicks

**Figure 2.**
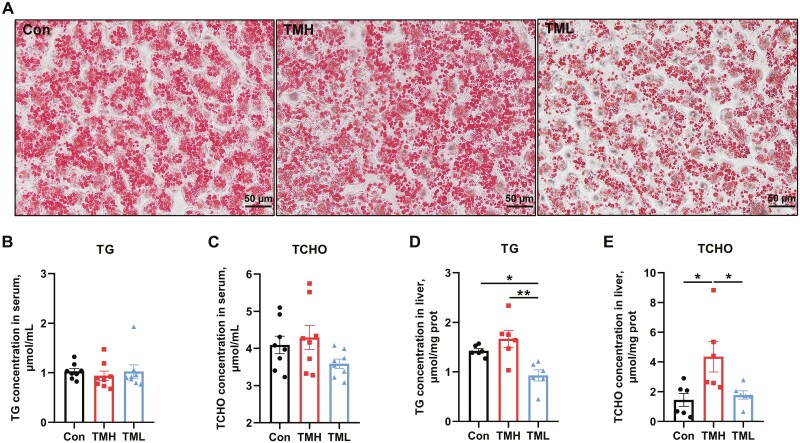
Effect of thermal manipulation with different temperatures on lipid content in serum and liver of chicks at 1 d of age. (A) Oil Red O staining of the liver (*n* = 6); (B) TG content in serum (*n* = 8); (C) TCHO content in serum (*n* = 8); (D) TG content in the liver (*n* = 6); (E) TCHO content in the liver (*n* = 6). Data are expressed as the mean ± SEM. *P < 0.05, **P < 0.01, ***P < 0.001.

As shown in Figure 2A, Oil Red O staining was used to evaluate the content of lipid droplets in the liver. A large accumulation of lipid droplets was observed in the control and TMH groups. However, sparse and irregular lipid droplets were observed in the TML group. The concentrations of TG and TCHO in serum and liver were further measured (Figure 2B to E). The results indicated that no changes in serum TG and TCHO levels were found (*P* > 0.05). In the liver, the TG content in the TML group was significantly lower than in the other 2 groups (*P* < 0.05), and TCHO was significantly lower than in the TMH group (*P* < 0.05). Furthermore, chicks in the TMH group had the highest TCHO levels compared to the other 2 groups (*P* < 0.05).

**Figure 3.**
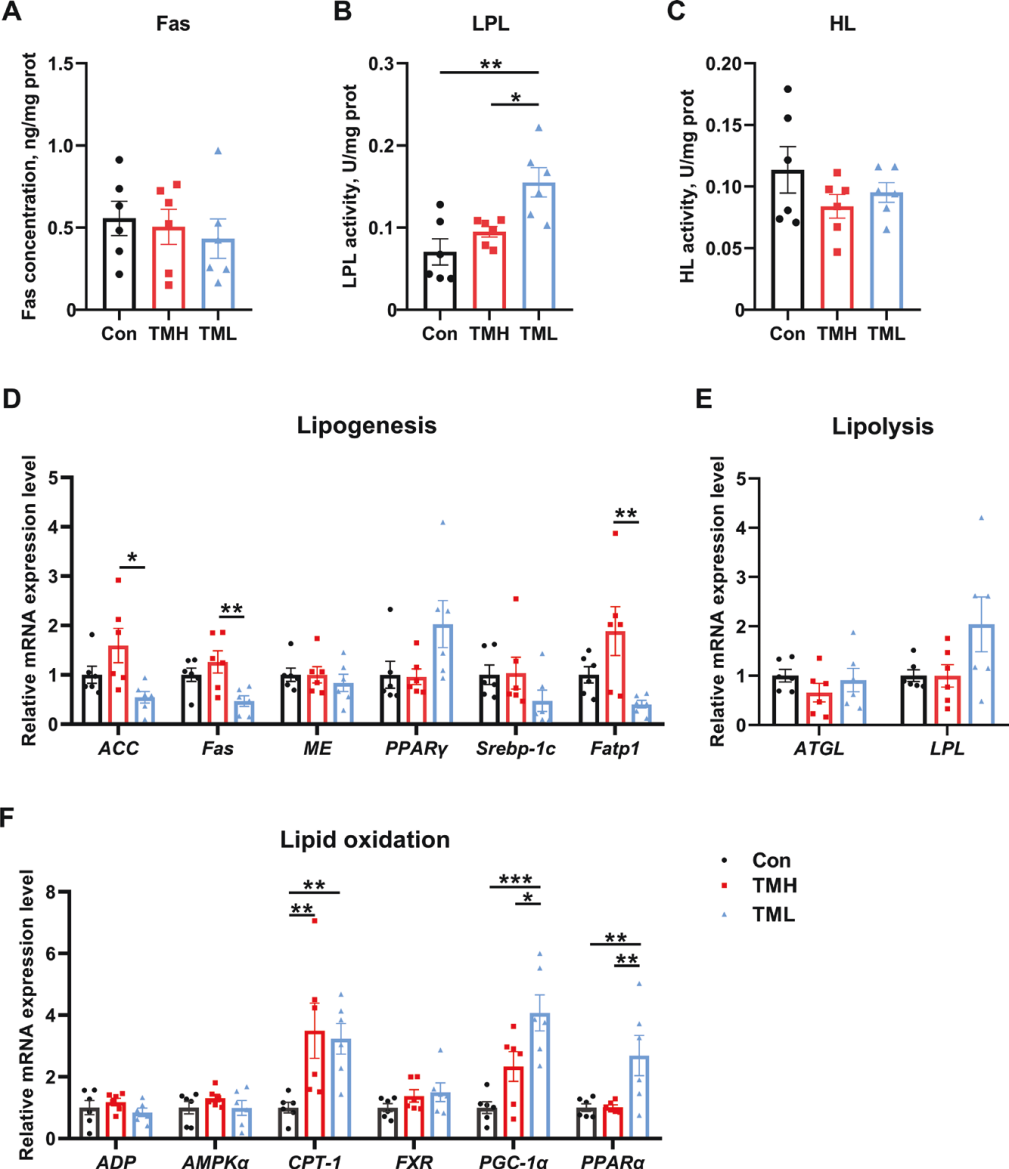
Effect of TM with different temperatures on lipid metabolism in the liver of chicks at 1 d of age. (A) Fas concentration in the liver (*n* = 6); (B) LPL activity in the liver (*n* = 6); (C) HL activity in the liver (*n* = 6); (D) the mRNA expression levels of genes associated with lipogenesis in the liver of chicks at 1 d of age (*n* = 6); (E) the mRNA expression levels of genes associated with lipolysis in the liver of chicks at 1 d of age (*n* = 6); (F) the mRNA expression levels of genes associated with lipid oxidation in the liver of chicks at 1 d of age (*n* = 6). Data are expressed as the mean ± SEM. *P < 0.05, **P < 0.01, ***P < 0.001.

Embryo TM did not yield significant alterations in Fas content or HL activity in the liver (Fig. 3A and C) (*P* > 0.05). However, LPL activity was notably increased (*P* < 0.05) in the TML group compared to the other groups (Fig. 3B). Compared with control, TMH and TML did not affect the expression of genes related to lipid synthesis (*P* > 0.05). Nonetheless, the expression levels of *ACC*, *Fas*, and *Fatp1* were lower (*P* < 0.05) in the TML group than in the TMH group (Fig. 3D). Additionally, there were no changes observed in the expression of lipolysis genes among the experimental groups (Fig. 3E). Compared with the control group, the expression of *CPT-1* in the TMH group, along with the expression of *CPT-1*, *PGC-1α* and *PPARα* in the TML group were all significantly upregulated (Fig. 3F) (*P* < 0.05). The levels of *PGC-1α* and *PPARα* expression in the TML group were also higher than in the TMH group (*P* < 0.05). There were no significant changes in the expression of other genes associated with lipid oxidation, including *ADP*, *AMPKα*, and *FXR*.

### Effects of TM on performance of chicks in early growth stages

Throughout the experiment, spanning from days 1 to 7, TMH and TML did not result in significant changes in average daily feed intake (**ADFI**) and average daily gain (**ADG**) among the experimental groups (Table 4) (*P* > 0.05). However, chicks in the TMH group exhibited a higher feed conversion ratio (FCR; ADFI/ADG) compared to those in the control and TML groups (*P* < 0.05). At 7 d of age, there were no changes in BW, or the percentages of liver, heart, breast muscle, thigh muscle, and abdominal fat among all groups (*P* > 0.05). The BL of chicks from the TML group showed a trend towards decrease compared to the other groups (*P* < 0.1).

**Table 4.** Effect of TM with different temperatures on performance of chicks at 7 d of age

Items	Con	TMH	TML	SEM	*P* value
ADFI (g/d)	10.21	11.18	10.62	0.241	0.2887
ADG (g/d)	3.06	2.70	2.96	0.084	0.2025
FCR (g/g)	3.35^b^	4.15^a^	3.58^b^	0.141	0.0239
BW (g)	60.48	59.66	57.45	0.688	0.1789
BL (cm)	19.17	19.58	18.84	0.131	0.0701
Liver/BW (%)	3.49	3.53	3.49	0.072	0.9654
Heart/BW (%)	0.97	0.87	0.86	0.024	0.1077
Breast muscle/BW (%)	5.27	5.26	4.86	0.103	0.1786
Thigh muscle/BW (%)	8.80	8.75	8.81	0.161	0.986
Abdominal fat/BW (%)	0.38	0.39	0.29	0.021	0.1124

^a,b^Values with different superscripts in the same row differ significantly (*P* < 0.05).

Con, control treatment (37.5 °C) during incubation; TMH, thermal manipulation with higher incubation temperature (39.5 °C and 65% RH for 8 h/d during 8th to 15th embryonic age) during incubation; TML, thermal manipulation with lower incubation temperature (20 °C and 65% RH for 1 h/d during 8th to 15th embryonic age) during incubation; ADFI, average daily gain; ADG, average daily gain; FCR, feed conversion ratio; BW, body weight; BL, body length.

### Effects of TM on hepatic lipid metabolism of chicks in early growth stages

**Figure 4.**
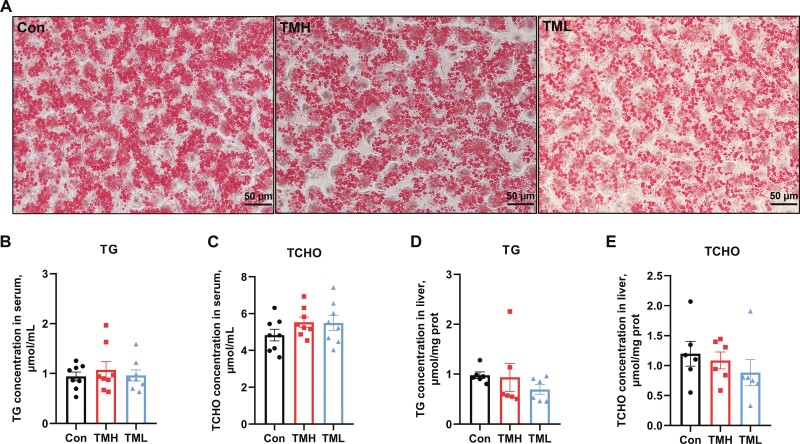
Effect of TM with different temperatures on lipid content in serum and liver of chicks at 7 d of age. (A) Oil Red O staining of the liver (*n* = 6); (B) TG content in serum (*n* = 8); (C) TCHO content in serum (*n* = 8); (D) TG content in the liver (*n* = 6); (E) TCHO content in the liver (*n* = 6). Data are expressed as the mean ± SEM. *P < 0.05, **P < 0.01, ***P < 0.001.

**Figure 5.**
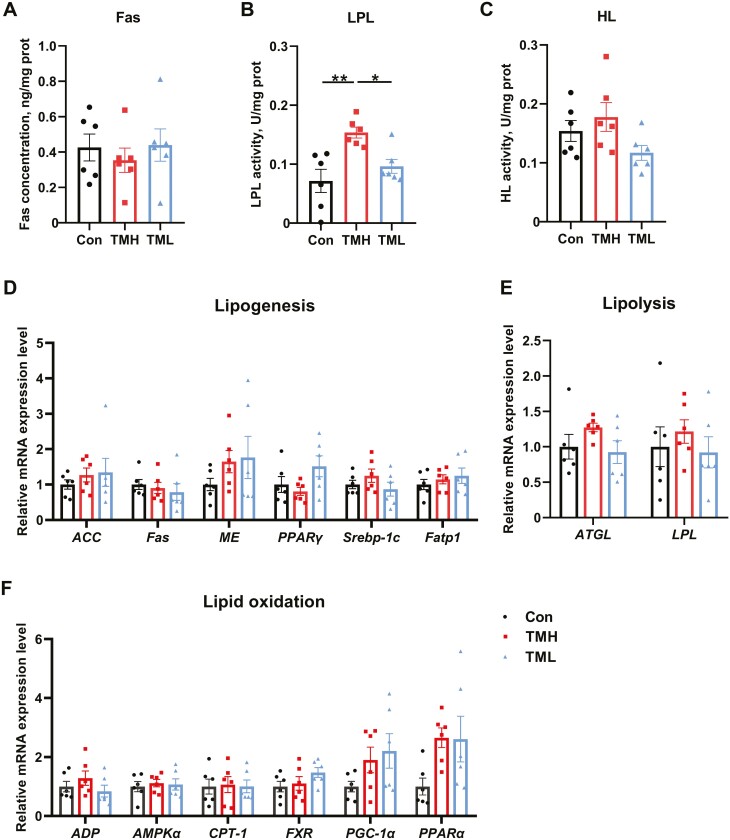
Effect of TM with different temperatures on lipid metabolism in the liver of chicks at 7 d of age. (A) Fas concentration in the liver (*n* = 6); (B) LPL activity in the liver (*n* = 6); (C) HL activity in the liver (*n* = 6); (D) the mRNA expression levels of genes associated with lipogenesis in the liver of chicks at 7 d of age (*n* = 6); (E) the mRNA expression levels of genes associated with lipolysis in the liver of chicks at 7 d of age (*n* = 6); (F) the mRNA expression levels of genes associated with lipid oxidation in the liver of chicks at 7 d of age (*n* = 6). Data are expressed as the mean ± SEM. *P < 0.05, **P < 0.01, ***P < 0.001.

As shown in Figure 4A, the accumulation of lipid droplets observed in the liver of the TML group appeared to be slightly lower compared to the other groups. However, there was no difference in TG and TCHO content in either serum or liver (Fig. 4B to E) (*P* > 0.05). Similarly, the Fas content and HL activity in the liver showed no differences among all groups (Fig. 5A and C) (*P* > 0.05). The activity of LPL in the TMH group was significantly increased compared to the control and TML groups (Fig. 5B) (*P* < 0.05). Moreover, TMH and TML had no effect on the expression of genes associated with lipid metabolism (Fig. 5D to F) (*P* > 0.05).

## Discussion

Incubation temperature is considered the most critical environmental factor affecting embryonic development and hatching efficiency. Slight changes in incubation temperature exert stress on the developing embryo and regulate overall development by altering embryo size, organ development, metabolic levels, and hatching ability (Yalcin et al., 2022). However, the effect of TM, whether through high or low temperatures, on embryonic growth is controversial due to variations in temperature, duration, and the embryonic stage of TM treatment (Goel et al., 2023). In the present study, the conditions for TM were selected based on both previous research and preliminary studies conducted in our lab. The temperature of 37.5 °C is widely recognized as the standard incubation temperature for chicken embryos, ensuring optimal development and hatchability. Higher incubation temperatures, such as 39.5 °C, have been employed in several studies to investigate their impact on embryonic development and post-hatch performance. For instance, previous research demonstrated that exposure to this temperature can induce beneficial physiological adaptations, including improved heat tolerance and altered metabolic rates (Yahav et al., 2004). Our choice was further supported by the absence of adverse effects on embryonic development observed in previous study with similar high-temperature (39 °C, 8 h/d) treatments (Han et al., 2022). However, extended low-temperature exposure is lethal to embryos. Interestingly, lower temperatures can impose a controlled stress without being lethal, provided the exposure duration is carefully managed (Afsarian et al., 2016). Our preliminary study indicated that a short-term exposure of 1 h to 20 °C did not significantly impair embryonic development (Han et al., 2022). Therefore, we selected a shorter duration for TML to prevent harmful effects while still assessing the potential benefits of TM.

In the past decade, many studies have highlighted that TM during the embryonic period is a significant means to influence the pre- and post-hatch development of chickens, potentially enhancing their environmental adaptability to resist heat or cold stress (Loyau et al., 2016; Madkour et al., 2022; Goel et al., 2023). Objective evidence in this study confirms that both TMH and TML can reduce the body temperature of chicks post-hatching (with TML showing only a downward trend). This suggests that TM may enhance the heat tolerance of layer chicks. However, before determining the effect of TM on later development and adaptability, hatchability is an important factor to evaluate the sensitivity of embryos to TM. In this study, high-temperature TM had no negative impact on hatchability and was expected to accelerate embryonic development. This finding is consistent with previous reports indicating that TM at 38.5 °C or 39.5 °C for 3 h from ED 16 to 18 did not alter hatchability (Yahav et al., 2004; Xu et al., 2023). Another report claimed that when eggs were incubated at 39.5 °C for 12 h from ED 7 to 16, hatchability was improved, but it was significantly reduced when the exposure was extended to 24 h (Piestun et al., 2008). In fact, in addition to the direct effect of embryonic age on hatchability, we speculate that the duration of TM exposure is also a critical factor. Intermittent TMH had no negative effect on embryonic development. Conversely, TML not only delayed the hatching process but also significantly reduced hatchability. This may indicate that chicken embryos are more sensitive to lower temperatures than to higher temperatures. The lower “Tona score” in the TML group also supports this view. Since this score considers chick activity, appearance, retracted yolk, eyes, legs, navel area, and remaining yolk to assess chick health, a lower score suggests that TM at 20 °C for 1 h may be too harsh for laying hen embryos (Tona et al., 2003). This finding aligns with our previous results that TML reduced hatchability, although chick quality was not affected (Han et al., 2022). It is worth noting that not all studies yield the same results. One study reported that TM at 15 °C for 30 min increased hatchability, and even extending the time to 60 min did not affect hatchability (Shinder et al., 2011). Another study reported that reducing the incubation temperature from 37.8 °C to 36.6 °C decreased hatchability by about 4% (Joseph et al., 2006). The exposure temperature and duration of TM may be the main reasons for these inconsistencies. Additionally, the complexity of embryonic development causes different responses to specific temperatures and durations, as well as varying environmental conditions. Therefore, the optimal period for TM treatment and the appropriate cold treatment temperature need further investigation for different strains.

Nutrient supply during the embryonic period depends on the yolk sac, with yolk lipids serving as the primary energy source during the latter half of incubation and early post-hatching (van der Wagt et al., 2020). Over 90% of the total energy produced by the embryo and the chick is derived from the oxidation of yolk lipids (Sato et al., 2006). Around ED 19, the residual yolk is internalized into the embryo’s abdominal cavity, and the rapid absorption of solids, particularly lipids, from the yolk is associated with active lipid metabolism in the embryonic tissues (Peebles et al., 2000). Thus, yolk sac absorption and lipid utilization are critical for both embryonic development and post-hatching chick quality (Yadgary and Uni, 2012). Importantly, metabolic rate, yolk utilization, and embryonic growth during hatching are temperature-dependent (Lourens et al., 2006). Higher incubation temperatures lead to an increase in the weight of unabsorbed residual yolk and, consequently, a decrease in the YFBM of the chicks after hatching (Molenaar et al., 2010). There is limited research on the effects of lower-than-standard incubation temperatures on embryonic development. A previous study reported that incubation temperatures of 35.6 °C or 36.7 °C starting at ED 15 did not affect residual YW at hatching compared to an incubation temperature of 37.8 °C (Maatjens et al., 2016). This may relate to the balance between metabolic rate and oxygen availability (Nangsuay et al., 2013). Our findings show that TMH did not affect post-hatching YW, BW, and YFBM. In contrast, although there were no significant differences in BW and YFBM, the increased residual YW in the TML group indicated delayed embryonic development. It is worth noting that the lower quality of chicks in the TML group was primarily due to unclosed navels and insufficient yolk sac absorption. This issue is likely related to the reduced relative weight of the liver. The liver, being the main tissue of lipid metabolism in poultry, converts yolk fatty acids into usable forms for the embryo (Speake et al., 1998). A reduction in the relative weight of the liver suggests developmental disorders in the chicks and contributes to the high residual YW.

In poultry, lipid metabolism and its regulatory mechanisms differ significantly from those in mammals. The liver is the primary organ of lipid synthesis, while lipogenesis in adipose tissue is limited. After hatching, the balance of lipid metabolism in poultry is maintained through the coordination of several processes, including lipogenesis, lipolysis, and lipid oxidation (Liu et al., 2019). We observed an increase in TCHO content in the TMH group, which may be attributed to the upregulation of the lipogenesis process. The liver features a de novo lipogenesis pathway that synthesizes triglycerides using non-lipid substances as substrates (Nematbakhsh et al., 2021). Fatty acid synthase is a crucial multienzyme for lipid synthesis, catalyzing the endogenous de novo synthesis of saturated fatty acids from simple molecular precursors such as acyl-CoA and malonyl-CoA, and subsequently transporting them to adipose tissue for storage (Hermier, 1997). Compared to the TML group, the higher levels of ACC and Fas in the liver of the TMH group indicate increased lipogenesis. Interestingly, chicks incubated at high temperatures may adapt to the hot environment by reducing their metabolism (Loyau et al., 2014; Goel et al., 2023). It has been reported that TM reduces the body temperature of broilers to mitigate heat stress injury (Collin et al., 2007; Al-Zghoul et al., 2019b). Consistent with this, we found that chicks in the TMH group had the lowest body temperature among all groups. Given the reduced demands for energy metabolism and thermogenesis, TMH appears to reduce lipid utilization, thereby facilitating greater lipid synthesis and storage (Sato et al., 2006).

In addition, the liver is the primary tissue for lipid oxidation and utilization. Triglycerides are hydrolyzed by lipolytic enzymes, including LPL and HL, to produce fatty acids, which are transported into the mitochondria by CPT-1 for oxidative phosphorylation and energy production (Wang and Eckel, 2009; Schlaepfer and Joshi, 2020). This process is positively regulated by various transcription factors, including peroxisome proliferator-activated receptor gamma coactivator 1-alpha (PGC-1α) and peroxisome proliferator-activated receptor alpha (PPARα) (Preidis et al., 2017; Cheng et al., 2018). In the current study, the increase in residual YW and the decrease in liver weight in the TML group indicated alterations in lipid metabolism. As suspected, the TML group exhibited lower TG levels and fewer lipid droplets in the liver, which correlated with increased lipolysis and lipid oxidation processes. These processes included enhanced LPL activity and elevated expression of CPT-1, PGC-1α, and PPARα. A major explanation for these observations is related to thermogenesis stimulated by low temperatures (Liang and Ward, 2006; Fedorova et al., 2022). As evidenced by the higher ΔT (changed rectal temperature from 1 to 7 d of age) in the TML group, chicks responded to the cold stimulate by increasing their metabolism to elevate body temperature. This increased energy requirement led to a shift in lipid metabolism toward oxidative energy supply.

Early embryonic stages may be impractical for TM, as even shorter durations can be more harmful to the developing embryo (Al Amaz and Mishra, 2024). Consequently, the optimal timeline for implementing TM strategies appears to be during mid- or late-embryonic stages. Studies have reported that embryos can respond to changes in incubation temperature at ED15 and ED20 (Tzschentke, 2008; Tong et al., 2013). This responsiveness is attributed to the maturation of thermoregulatory abilities, which act as a negative feedback mechanism to adjust heat production and metabolic activities in response to TM (Tzschentke, 2008). Coincidentally, the lipid metabolism capacity of liver also gradually improves around ED15. Given that yolk lipids serve as the primary energy source during the mid- and late-embryonic stages, and lipid metabolism plays a crucial role in thermogenesis and growth development (Wong and Uni, 2021), it can be inferred that the effect of TM on growth depends on changes in liver lipid metabolism, which is regulated by the thermoregulatory system.

However, the effects of both TMH and TML on liver lipid metabolism appear to be confined to the embryonic period. This conclusion is based on the comparative analysis of data collected at these 2 time points. Specifically, we noted that liver TG content and related gene expressions, which were significantly affected by TM at day 1, showed no significant differences by day 7. This temporal attenuation of effects suggests that the initial impact of TM does not persist as the chicks continue to grow. This result contradicts a previous study indicating that high-temperature treatment had a more significant impact on liver lipid metabolism in ducks after hatching rather than during the embryonic period (Wang et al., 2014). Given the differences in breeds and exposure times, direct comparisons between the 2 studies are not meaningful. In fact, numerous studies have reported the long-term effects of TM, such as enhancing skeletal muscle development (Piestun et al., 2013), regulating nutrient digestion and absorption capabilities (Al-Zghoul et al., 2019a), and mitigating heat stress-induced intestinal inflammation in broiler chickens during the late growth period (Xu et al., 2023). Our study showed that TM had no significant impact on the growth performance and liver lipid metabolism of layer chicks in the early growth period, which may indicate that the effects of TM are not long-lasting (Collin et al., 2007). The diminished influence of TM as chicks grow can be attributed to several factors related to physiological and metabolic adaptations. During the early stages of development, embryonic and neonatal chicks are highly sensitive to environmental conditions, including temperature (Yalcin et al., 2022). TM during these stages can induce immediate and significant metabolic changes, particularly in processes such as lipid metabolism. However, as chicks continue to grow and develop, their physiological systems become more robust and capable of maintaining homeostasis despite environmental variations. This increased resilience likely reduces the relative impact of earlier TMs. Additionally, the initial metabolic changes induced by TM, such as alterations in lipid metabolism, may be part of a transient adaptive response designed to optimize immediate survival and growth rather than long-term performance.

Furthermore, as chicks mature, they undergo numerous developmental changes, including improvements in thermoregulation and metabolic efficiency (Tzschentke, 2008). These changes may override or compensate for the early influences of TM, leading to a reduction in observable effects. It is important to consider the overall metabolic adjustments in response to TM. While TMH may improve the heat tolerance of chicks, the metabolic shifts required to cope with increased incubation temperatures might result in a temporary inefficiency in energy utilization, reflected as a higher FCR at this early stage. However, this hypothesis lacks sufficient data for support, and further research is needed to investigate changes in energy metabolism balance and mitochondrial function. Additionally, it is also possible that post-hatching nutrition and environmental conditions play a significant role in modulating the long-term outcomes of early-life thermal experiences (Goel et al., 2023). However, it is important to note that this study did not involve extreme environmental challenges. TM emphasizes temperature adaptability (Loyau et al., 2016), so evaluating changes in lipid metabolism during heat stress may be more relevant for production purposes. Additionally, considering that laying hens have a longer growth cycle and more active liver lipid metabolism during the laying period, further exploration is needed to determine whether TM has a positive effect in these contexts.

## Conclusion

In summary, embryo TM with either higher temperature (39.5 °C, 8 h/d) or lower temperature (20 °C, 1 h/d) during 8th to 15th embryonic age can affect the development and hepatic lipid metabolism in layer-type chick after hatching. The impact of the low incubation temperature was more pronounced, with even short-term TML exposure being sufficient to reduce hatchability and chick quality, as well as to decrease liver fat accumulation by enhancing lipolysis and lipid oxidation. However, these effects diminished progressively as the chicks developed.

## Data Availability

All data generated or analyzed during this study are available from the corresponding author upon reasonable request.

## Abbreviations

ACC: acetyl-CoA carboxylase
ADFI: average daily feed intake
ADG: average daily gain
ADP: adiponectin
AMPKα: adenosine 5ʹ-monophosphate (AMP)-activated protein kinase alpha
ATGL: adipose triglyceride lipase
BL: body length
BW: body weight
CPT-1: carnitine palmitoyltransferase-1
ED: embryonic day
Fas: fatty acid synthase
Fatp1: fatty acid transport protein 1
FCR: feed conversion ratio
FXR: farnesoid X receptor
HL: hepatic lipase
RH: relative humidity
LPL: lipoprotein lipase
ME: malate synthase
PGC-1α: peroxisome proliferator-activated receptor gamma coactivator 1-alpha
PPARα: peroxisome proliferator-activated receptor alpha
PPARγ: peroxisome proliferator-activated receptor gamma
Srebp-1c: sterol regulatory element-binding protein 1c
TCHO: total cholesterol
TG: triglyceride
TM: thermal manipulation
TMH: thermal manipulation with high temperature
TML: thermal manipulation with low temperature
YFBM: yolk-free body mass
YW: yolk weight
